# A case of nodular fasciitis that was difficult to distinguish from sarcoma

**DOI:** 10.1016/j.ijscr.2019.10.036

**Published:** 2019-10-22

**Authors:** Hisato Nagano, Tomoharu Kiyosawa, Shimpo Aoki, Ryuichi Azuma

**Affiliations:** Department of Plastic and Reconstructive Surgery, National Defense Medical College Hospital, Tokorozawa, 359-8513, Saitama, Japan

**Keywords:** Case report, Histopathological diagnosis, Nodular fasciitis, Malignant peripheral nerve sheath tumor, Sarcoma, Spontaneous regression

## Abstract

**Introduction:**

Nodular fasciitis is a reactive proliferative lesion. It is often difficult to distinguish from sarcoma, such as malignant peripheral nerve sheath tumor (MPNST). Therefore, both pathological findings and clinical features must be considered.

**Presentation of case:**

A 75-year-old man presented with a 1 × 1 cm mass on his left forearm that had enlarged rapidly without specific symptoms. Magnetic resonance imaging revealed a mass adjacent to brachioradialis. The lesion showed iso-intensity to muscle on T1-weighted images and hyperintensity on T2-weighted images. We made a clinical diagnosis of nodular fasciitis. Under observation, the mass gradually decreased in size and disappeared after two months. Four years later, another lesion appeared at the same site. We performed total excisional biopsy and histopathological examination indicated low grade MPNST or recurrent nodular fasciitis. Since malignancy could not be excluded, wide resection was performed. At 4 years postoperatively, there has been no recurrence.

**Discussion:**

In this patient, nodular fasciitis of the right forearm was diagnosed clinically and showed spontaneous regression. However, recurrence was noted after four years. While the clinical features suggested recurrent nodular fasciitis, pathological findings indicated the possibility of low grade MPNST.

**Conclusion:**

When it is difficult to determine whether a lesion such as nodular fasciitis is benign or malignant, the patient should be managed by considering the possibility of malignancy.

## Highlights

• Nodular fasciitis is difficult to distinguish from sarcoma.
• Clinical and pathological findings may not correspond, as in our case.
• If sarcoma cannot be excluded, treatment should consider the risk of malignancy.

## 1 Introduction

Nodular fasciitis is a reactive proliferation of fibroblasts/myofibroblasts in the fascia and subcutaneous tissue. Histological examination sometimes reveals cellular heterotypia and polymorphism. Therefore, nodular fasciitis is often misdiagnosed as soft tissue sarcoma. Immunohistochemistry is useful for diagnosis, but there are only a few markers for malignant peripheral nerve sheath tumor (MPNST). If immunohistochemical findings are unclear, differential diagnosis is difficult. Also, the clinical and histological features sometimes do not correspond, and making a definite diagnosis can be challenging.

We report a patient who presented to a tertiary hospital in Japan with a mass that was clinically diagnosed as nodular fasciitis and showed spontaneous regression. Four years later, recurrence was noted. Histopathological examination suggested low grade MPNST, although clinical findings were consistent with recurrent nodular fasciitis. This case was reported in line with the SCARE criteria [1].

## 2 Presentation of case

**Fig. 1:**
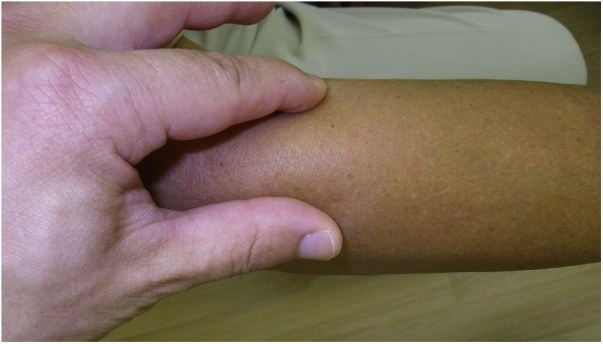
Appearance of the initial tumor A subcutaneous mass (10 × 10 mm) in the right forearm was clinically diagnosed as nodular fasciitis.

A 75-year-old man presented to the outpatient department with a 1 × 1 cm mass on his left forearm that had enlarged rapidly over the previous 2 weeks without specific symptoms (Fig. 1). His past history included Dupuytren's contracture of the hand and sick sinus syndrome. He was on no medications and his family history was non-contributory. On examination, he appeared well with normal vital signs. There were no significant findings, except for a mass on his left forearm. The mass was firm, dome-shaped, and adherent to brachioradialis muscle, but not the overlying skin. Laboratory test results and urinalysis were unremarkable. Magnetic resonance imaging revealed a mass measuring 9 × 6 mm adjacent to brachioradialis. The lesion showed iso-intensity to muscle on T1-weighted images and hyperintensity on T2-weighted images, as well as contrast enhancement. From these findings, we made a clinical diagnosis of nodular fasciitis. Excisional biopsy was not performed. Under observation, the mass gradually decreased in size and disappeared after two months.

**Fig. 2:**
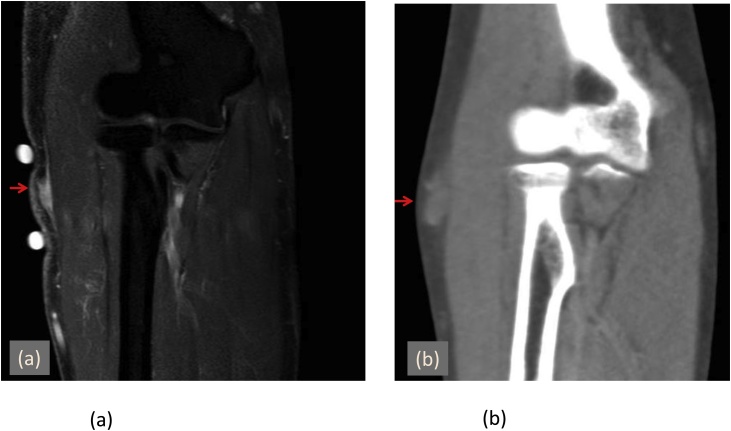
Comparison of the initial and recurrent tumors. (a) MRI showed a mass (9 × 6 mm) that was thought to be nodular fasciitis. (b) Four years later, the recurrent subcutaneous mass (23 × 10 mm) was difficult to diagnose by CT.

Four years later, another lesion appeared at the same site. Contrast computed tomography (CT) demonstrated a mass measuring 23 × 10 mm (Fig. 2). We considered the possibility of recurrent nodular fasciitis or another tumor and performed total excisional biopsy under general anesthesia.

**Fig. 3:**
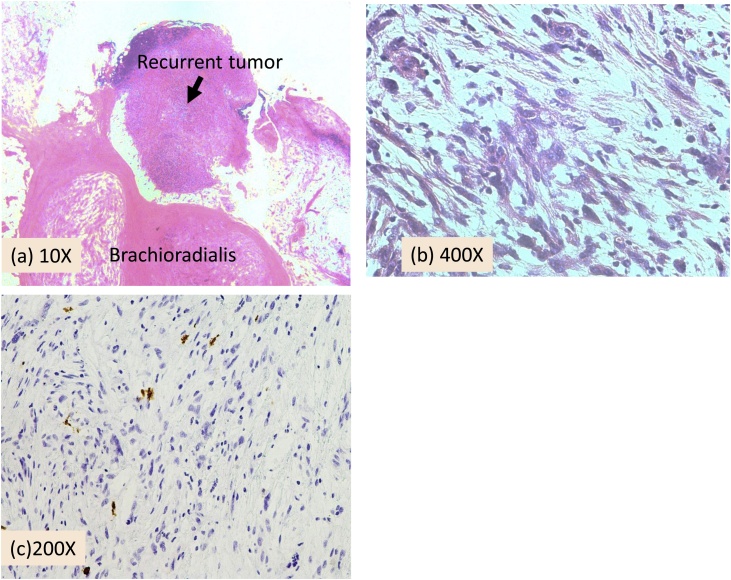
Pathological findings of the recurrent tumor. (a) The mass is surrounded by a well-defined fibrous capsule. (b) The tumor cells have eosinophilic cytoplasm and polymorphic nuclei with anisokaryosis. Physaliphorous cells are sometimes seen in association with proliferation of fibroblasts and capillaries. Cells are also seen proliferating in the interstitial region or form irregular bundles in myxoid stroma. (a, b: hematoxylin-eosin stain). (c) Immunostaining demonstrates that almost all of the tumor cells are negative for S100 protein.

Histopathological examination revealed spindle-shaped polymorphonuclear cells with eosinophilic cytoplasm and vacuoles (Fig. 3). The tumor cells formed multiple irregular bundles and were arranged in a storiform pattern. Both hypercellular and hypocellular areas were noted with myxomatous stroma. There were 3 mitoses per 10 high power fields in the hypercellular areas. No necrotic areas were seen. Immunohistochemistry was negative for CAM5.2, epithelial membrane antigen, alpha-smooth muscle actin (αSMA), desmin, and CD34. In addition, S-100 was almost negative. We discussed the patient with sarcoma specialists at a multidisciplinary meeting to make a final diagnosis. Combining the histological and clinical findings, it was considered that the tumor could be a low grade MPNST or recurrent nodular fasciitis.

**Fig. 4:**
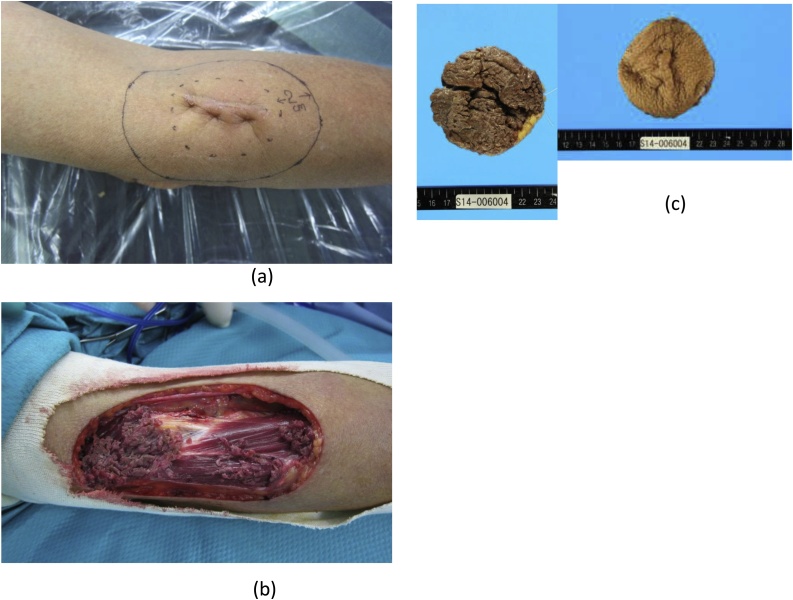
Additional excision. (a) The lateral surgical margin was set at 20 mm. (b) The deep margin was set at the full thickness of brachioradialis muscle. (c) Residual tumor was not detected either macroscopically or histopathologically.

Since malignancy could not be excluded, wide resection was performed with a lateral margin of 20 mm from the previous surgical scar and partial removal of brachioradialis muscle at the deep margin (Fig. 4). The wound was covered with artificial dermis and a full thickness skin graft was applied after 5 weeks. Surgery was performed by a board certified fellow of the Japan Society of Plastic and Reconstructive Surgery. Histopathological examination showed reactive proliferation of fibroblasts and no atypical cells. There were multinucleated cells and proliferating cells in the interstitial region along with myxomatous change. Immunohistochemistry was positive for vimentin and partially positive for αSMA. However, it was negative for desmin, h-caldesmon, CD31, CD34, S-100, cytokeratin, and β-catenin. The patient was followed at our outpatient clinic every 3 months and underwent annual CT scanning. At 4 years postoperatively, there has been no recurrence and the patient has no sensory or motor dysfunction. The patient provided informed consent for the details of this case to be reported.

## 3 Discussion

Nodular fasciitis is a benign reactive lesion that often grows rapidly over several weeks before the patient presents with a mass [2,3]. It was first described by Konwaler [4] in 1955 as subcutaneous pseudosarcomatous fibromatosis. The mass is usually less than 4 cm in diameter [3] and 71% are smaller than 2 cm [5]. Nodular fasciitis may occur anywhere on the body, but most commonly affects the forearm (27–29%) [2]. It sometimes shows spontaneous regression [6] and recurrence is rare, even after incomplete excision [2], with no reports of malignant transformation. These lesions may show atypia and mitoses, making nodular fasciitis difficult to distinguish from sarcoma [5,[7], [8], [9]]. In fact, Plaza et al. [10] reported that two thirds of their cases were misdiagnosed as sarcoma. The etiology is unknown, but it is thought that trauma or infection may promote development of nodular fasciitis. Conservative treatment is generally recommended, although some authors have advocated excision [11,12].

MPNST is a neurogenic sarcoma, and about 50% of patients have neurofibromatosis type 1. In sporadic cases, MPNST often arises from a large nerve such as the sciatic nerve [13]. MPNST usually presents as a painless mass with numbness in the territory of the affected nerve. The lesion often exceeds 5 cm in diameter at the time of diagnosis [13]. It is unknown whether these tumors are derived from a specific cell type [14]. MPNST is generally treated by wide resection, while chemotherapy has a limited effect [15]. There have been some reports about the possible effectiveness of radiation therapy [16], but this is uncertain.

Our patient’s tumor was 2 cm in diameter, which is typical of nodular fasciitis and rather small for MPNST. Spontaneous regression is a feature of nodular fasciitis, but not MPNST. Also, our patient had no history of neurofibromatosis. Moreover, the tumor did not clearly arise from a nerve, suggesting it was not MPNST. Based on these points, our clinical diagnosis of nodular fasciitis seems correct.

On MR imaging, nodular fasciitis shows a homogeneous low signal intensity on T1-weighted images and a heterogeneous intermediate signal intensity on T2-weighted images, with surrounding edema and slightly inhomogeneous enhancement [17]. It can also demonstrate aggressive features such as transcompartmental spread and osseous or intra-articular involvement [17]. The imaging findings are generally nonspecific [17]. Accordingly, it is often difficult to separate nodular fasciitis and sarcoma based on imaging data [17,18]. Therefore, nodular fasciitis is generally distinguished from sarcoma by immunohistochemical examination. However, diagnosis of MPNST is difficult due to lack of specific immunohistochemical markers. Although S-100 is a useful marker for MPNST [19], about one-third of these sarcomas are negative for S-100 [20].

In our patient, S-100 staining was negative in most of the tumor and was weakly positive in some areas. While pathological findings tended to suggest MPNST, the clinical course supported a diagnosis of nodular fasciitis. Making a definite diagnosis was impossible because it was difficult to decide whether S-100 staining was positive or negative.

If making an accurate diagnosis from clinical findings is difficult, the surgeon often cannot clearly exclude “possible sarcoma”. It is usual to place more weight on the pathological findings in such cases. If the clinical course does not fit the pathological diagnosis, the final diagnosis should be made by integrated assessment of all available data. Chromosomal analysis and genetic testing are performed for research, but are generally unavailable in routine practice. When it is difficult to determine whether a lesion is benign or malignant, management should be based on the possibility of malignancy.

## 4 Conclusion

We reported a rare case of clinical nodular fasciitis of the right forearm showing spontaneous regression and recurrence after four years. While the clinical features suggested recurrent nodular fasciitis, pathological findings were consistent with low grade MPNST. When clinical and pathological features differ, the final diagnosis should be based on comprehensive assessment. However, reaching a definite conclusion may sometimes be impossible.

## Funding

There are no sources of funding to declare.

## Ethical approval

A case report is exempt from ethical approval in our institution.

## Consent

We obtained consent to publish a case report from the patient.

## Author contribution

HN drafted the article. TK, SA and RA had revised the manuscript critically. TK had revised the histopathological findings. All authors contributed to study concept or design at this submission and approved the final version.

## Registration of research studies

N/A.

## Guarantor

Corresponding author; Hisato Nagano.

## Provenance and peer review

Not commissioned, externally peer-reviewed.

## Declaration of Competing Interest

There are no conflict of interests to declare.
